# Nævus, or the Effects of Dental Operations upon the Female during the Period of Utero-Gestation

**Published:** 1878-11

**Authors:** E. S. Chisholm

**Affiliations:** Tuscaloosa, Ala.


### NÆVUS, OR THE EFFECTS OF DENTAL OPERATIONS UPON THE FEMALE DURING THE PERIOD OF UTERO-GESTATION.

BY DR. E. S. CHISHOLM, TUSCALOOSA, ALA.

Owing to a case of harelip malformation and fissure of the gum of a newly born infant, resulting, as I suppose, from the filling of a tooth for the mother while pregnant, I decided to offer a few thoughts upon diagnosis, or rather the importance of correct diagnosis of those diseases and sympathetic affections incident to pregnancy.

The word, diagnose, is derived from the Greek, signifying, "I know," and is that part of medicine whose object is the discrimination of disease, the knowledge of the pathognomonic signs of each, and as Dunglison says, "is one of the most important branches of general pathology."

As this subject affords such a wide field of reflection and study, I will confine my essay to the discussion of but one of its branches, the diagnosis of those diseases and sympathetic affections that accompany pregnancy, and then offer some suggestions as to our duties as dentists toward the pregnant female who applies to us for relief from some of the many ills to which she has fallen heir.

I have often been asked why it is that diseases of the uterus are so much more common now than in former times, and you will occasionally meet with good old grandmothers who will shrewdly remark, "Why, Doctor, when I was young I never heard of ladies having these complaints, what is the reason we hear so much of them now?" This question is readily answered. It is not a necessary sequitur that because diseases of the uterus were not recognized they did not exist. These affections, although no doubt much enhanced by the increasing neglect of the general ordinances of health, are of no recent date, but on the contrary have formed their part in the catalogue of human suffering and death from the earliest periods of creation.

The machinery of the physical world and the revolutions of the sun were no less perfect thousands of years ago than they are at present, yet how profoundly ignorant was man of their true nature; how inadequate to explain what then appeared to him mysteries beyond the ken of human intelligence. But these mysteries have now become universal truths; they have yielded to the progress of science, are perfectly understood, and constitute the every day lessons of the school room.

The ample means, therefore, which we now possess of investigating uterine disorders, and the comparative facility with which the true nature of these diseases is arrived at, give to this class of maladies an identity which formerly did not belong to them; and hence, what in the remote periods of the science of physic were regarded as idiopathic affections of the head, chest, abdomen, etc., are now recognized to be symptomatic disturbances, or mere effects of disease in the uterine organs.

The object proper of this paper is to impress strongly upon the minds of our junior members particularly, the importance of a thorough course of study of this important subject. We should be prepared to diagnose most of the cases that come before us, and I assure you my young brethren, that the pregnant female will often become your patient, for toothache, one of the most prominent symptoms of pregnancy, will cause her to apply to you for relief. And when she does come, then it is, you should have in store all the information that can be gathered on the subject. You should be able to decide intelligently whether the toothache be a disease or a symptom. The examination should be conducted in that chaste and delicate manner that should characterize a gentleman dentist, but nevertheless the examination should be made. No hasty conclusions should be jumped at, but we should have such command of the subject that we may know where to begin and where to end, and not tire our patient with useless repetitions. There should be no display of instruments, no offensive odors in our offices; no skulls or unsightly objects of any kind, for the extreme impressibility of the nervous system of our patient teaches us the necessity of preventing them from witnessing scenes that are in any way repulsive to them, for although no injury may thereby be done to the child, the mind of the mother may remain much troubled with anticipations of some deformity.

There is no denying the fact, however, that deformity and "mother marks" may be caused by external causes, such as fright, any sudden shock either physical or mental, and we are satisfied that sometimes the very fear upon the part of the mother, that some terrible deformity or mark has been fastened upon her child from beholding some disgusting or repulsive object, may produce the very calamity for which her fears were aroused.

While engaged in the practice of medicine, on two or three occasions, females intimated to me that they felt strong-y impressed that their child when born would be marked, they having allowed to go unsatisfied a longing for cherries, peaches or some other article of food, perhaps out of season at the time, and to my astonishment the mark was found just where the mother declared it would be.

I confess that the subject of mother marks has never been satisfactorily explained in our medical works, and some of our over wise men and fluent writers ridicule the idea of any such thing. But there is one proposition that can not be denied, that in all ages of the world's history, nature has at times deviated from the proper performance of its functions in the reproduction of the human as well as the animal species, resulting in monstrosities, harelip, club foot and a long list of abnormal conditions, rendering the unfortunate victim of either not only a mark for the public gaze, but an object of sympathy, yea, even of loathing by every beholder and alike repulsive to the poor blighted creature itself.

These anomalies come under the head of congenital or connate diseases. Congenital meaning diseases or faulty conformation which children have at birth; while connate, having almost the same signification, more particularly applies to those diseases or affections that may have supervened during gestation or delivery.

This is certainly a subject that should engage the attention of all classes of intelligent people as well as the dentist. If Jacob of old could by setting striped poles in the midst of his flocks produce lambs that were ringed, streaked and striped, the same natural causes that operated then may operate now.

I have long since felt that this subject in its different bearings upon the future race of man has not been appreciated and therefore under estimated by all classes of society.

A poor dejected woman in abject poverty, maltreated by a besotted husband, can not reasonably be expected to bring forth a child as perfectly developed in its mental faculties as one who is surrounded by all that is calculated to render her cheerful, contented and happy. This idea may appear novel to some of you, but in my opinion it is nevertheless tenable ground upon which to base a theory.

There is no denying the fact that the characteristics of the mother more often than those of the father are impressed upon the child, and it is the duty of the husband to place before the companion of his bosom, so far as is in his power to do so, while she is "encient," everything that is pleasing to her senses, for just in proportion as she passes the long wearisome months of her pregnancy, resigned cheerfully to her lot, fondly anticipating the happiness in store for her at the end of her term, just in the same proportion will her offspring partake of her nature. If she be cheerful and happy, her child will be sprightly; but if she pass the time with evil forebodings, afraid to look at any ordinary object for fear of marking her child, ever looking at the dark side of the picture and repelling every ray of hope and sunshine that may seek to enter her breast, her child will make just such an one as we often see, spleenful, spiritless, suspicious, and having inherited an evil nature it is apt to cling to the unfortunate creature through life. Not only our disposition but our sins too are visited upon our children even to the third and fourth generation. Woman, then, is a wonderful organism, through whom may be transmitted our virtues as well as our sins, and we may add that through our ignorance or our temerity as dentists, we are liable to fasten a deformity upon the yet unborn infant that would blast its happiness for life, and be a lasting monument to our shame.

The case of hare lip and shocking deformity referred to as the text for my essay, occurred in Georgia not long since, and is well authenticated. When the mother of the child was but a few weeks "gone, as the ladies term it, she went to a dentist and had an upper bi-cuspid tooth filled. The operation was a painful one and the tooth felt uneasy for a long time thereafter, as the patient informed me. In due time she was delivered of her child, and to her and her husband's mortification and surprise, there was an extensive fissure through the lip and through the alveolar ridge and gum, on the same side of the mouth, and in the same position of the tooth that had been filled. They were both impressed at once with the belief that the mal formation was caused by the filling of the tooth. From all the light before me I am inclined to the same opinion. When, therefore, a patient who is "encient" comes to us for relief from toothache, or to have operations performed upon her teeth, we should at once study her temperament. If she possesses that strong will, coupled with that heroic power of endurance and fortitude that many women manifest when subjected to pain, we need not hesitate even to extract her tooth, though this should not be done if it can well be avoided, Palliatives should first be used; should they fail, without making a display of instruments, I would engage her in pleasant conversation, leading her mind away from her present suffering, assuring her that it will be but a trifling operation, if there be any warrant whatever for such an assertion, and when I consider her as prepared for the operation, I select such instrument as is necessary, and in a deliberate manner proceed to extract the tooth, without lancing the gum. Just so soon as the tooth is out I remind her of the pleasant nights rest in store for her, and the improvement there will be in her feeling generally now that the offending tooth is removed.

On the other hand, if the patient comes with all her grandmothers, and a score of Job's comforters along with her, each one in her presence asking me of the danger that might ensue from extracting the tooth, I promptly advise that we try every other expedient before a final resort to extraction.

On one occasion I stepped into a brother dentist's office to make a social call, my ears, instead of being regaled with the sound of pleasant conversation or the busy tick of the mallet, heard the sobs and bemoanings of a lady patient for whom the Doctor was preparing a cavity for filling. The decay was upon the labial surface of a lower incisor, running around the margin of the gum. All dentists know that these are the most excruciatingly sensitive cavities of any in the mouth. Our brother, I must say, is a fine operator, and, as is characteristic of him, was making the chips fly, and the patient was letting the tears flow in profusion. She was an intelligent lady and exhibited by her manner and suppressed emotion that she was doing all in her power to bear the painful operation without a murmur, but the pain was more than she could bear; she could suppress her feelings no longer, and exclaimed, "Doctor, please extract the tooth or let it alone and allow it to decay away, I will not be tortured thus to save it." The Doctor called me to the chair and asked my advice as to what should be done. I suggested the applicatien of Chlo zinc or some other pain obtunder. He threw up his hands and at the top of his voice cried out, "Why, bless your soul, I never once thought of it." He made the application, excavated the cavity with comparative comfort to the patient. When he had finished the operation, his patient turned to me and said, "I will remember you with kindness so long as I live."

Now, my brothers, let us learn a lesson from this narative. Let us strive to inflict as little pain as possible, not only upon the patient who is "in a delicate condition," but all who confidingly place themselves in our power. Let us never have to reproach ourselves after committing an error, by the acknowledgment that we knew better, but really did not think of the proper course to pursue. Let us so act toward our patients that they will ever be ready to say, "I will remember you kindly so long as I live."

I might prolong this essay almost indefinitely in detailing the terrible consequences that might result to mother and child by indiscrimination upon our part in the treatment of our patients. But time with its rapid strides has whirled around another year, and another meeting of our Association, and as is the rule with most of us, have seized upon the last few hours of the departing year to prepare a paper for our meeting. If, however, I have succeeded in arousing a spirit of inquiry in the minds of those for whom these hints are more particularly directed, I shall feel well compensated for the time and labor bestowed in its preparation.

